# Supplementary material for: Risk of gastrointestinal cancer in patients with an elevated level of gamma-glutamyltransferase: A nationwide population-based study
Source: PLoS One. 2021 Feb 5;16(2):e0245052. doi: 10.1371/journal.pone.0245052 (PMC7864398; doi:10.1371/journal.pone.0245052)
Supplement: S1 Table — (DOCX) [file pone.0245052.s001.docx]

**S1 Table. Baseline characteristic by serum GGT level in men (*N* = 4,234,414)**

|  | **GGT quartile** | | | | ***P*-value** |
| --- | --- | --- | --- | --- | --- |
|  | **Q1** | **Q2** | **Q3** | **Q4** |  |
|  | ***N* = 1,047,272** | ***N* = 1,026,998** | ***N* = 1,105,896** | ***N* = 1,054248** |  |
| **Age (years)*** | 43.82 ± 15.11 | 45.24 ± 14.06 | 45.93 ± 13.05 | 45.89 ± 11.88 | <.0001 |
| **Waist circumference (cm)*** | 79.55 ± 7.03 | 82.15 ± 7.29 | 84.54 ± 7.37 | 86.49 ± 7.50 | <.0001 |
| **Body mass index (kg/m^2^)*** | 22.68 ± 2.60 | 23.62 ±2.78 | 24.55 ± 2.90 | 25.31 ± 3.09 | <.0001 |
| **Exercise (%)** | 608,186 (58.07) | 600,209 (58.44) | 645,502 (58.37) | 611,610  (58.01) | <.0001 |
| **Smoking status (%)** |  |  |  |  | <.0001 |
| **Non-smoker** | 415,350 (39.66) | 350,730 (34.15) | 330,042 (29.84) | 266,567 (25.29) |  |
| **Ex-smoker** | 240,158 (22.93) | 252,006 (24.54) | 277,411 (25.08) | 244,539 (23.20) |  |
| **Current smoker** | 391,764 (37.41) | 424,262  (41.31) | 498,443 (45.07) | 543,142 (51.52) |  |
| **Low income (%)** | 243,744 (23.27) | 224,136 (21.82) | 232,773 (21.05) | 223,847 (21.23) | <.0001 |
| **Drinker (%)** | 551,288 (52.65) | 618,124 (60.19) | 744,621 (67.33) | 817,513 (77.54) | <.0001 |
| **Hypertension (%)** | 170,168 (16.25) | 220,127 (21.43) | 301,020 (27.22) | 366,068 (34.72) | <.0001 |
| **Dyslipidemia (%)** | 77,680  (7.42) | 128,740 (12.54) | 203,195 (18.37) | 272,423 (25.84) | <.0001 |
| **Metabolic syndrome (%)** | 111,188 (10.62) | 188,958  (18.40) | 314,293 (28.42) | 441,012 (41.83) | <.0001 |
| **Fasting glucose (mg/dl)*** | 93.16 ± 18.44 | 95.84 ± 20.95 | 98.81 ± 24.13 | 103.7 ± 28.56 | <.0001 |

GGT, gamma-glutamyltransferase.

*Value was presented with mean ± SD.
