# Supplementary material for: Risk of gastrointestinal cancer in patients with an elevated level of gamma-glutamyltransferase: A nationwide population-based study
Source: PLoS One. 2021 Feb 5;16(2):e0245052. doi: 10.1371/journal.pone.0245052 (PMC7864398; doi:10.1371/journal.pone.0245052)
Supplement: S2 Table — (DOCX) [file pone.0245052.s002.docx]

|  | **GGT quartile** | | | | ***P*-value** |
| --- | --- | --- | --- | --- | --- |
|  | **Q1** | **Q2** | **Q3** | **Q4** |  |
|  | ***N* = 1,047,272** | ***N* = 1,026,998** | ***N* = 1,105,896** | ***N* = 1,054248** |  |
| **Age (years)*** | 43.67 ± 14.34 | 46.65 ± 14.62 | 50.03 ± 14.34 | 53.09 ± 12.95 | <.0001 |
| **Waist circumference (cm)*** | 72.81 ± 7.71 | 74.47 ± 8.23 | 76.90 ± 8.76 | 80.13 ± 9.11 | <.0001 |
| **Body mass index (kg/m^2^)*** | 22.03 ± 2.78 | 22.57 ± 3.04 | 23.42 ± 3.31 | 24.60 ± 3.56 | <.0001 |
| **Exercise (%)** | 428,436 (44.90) | 443,919 (44.29) | 428,981 (43.18) | 389.074 (41.55) | <.0001 |
| **Smoking status (%)** |  |  |  |  | <.0001 |
| **Non-smoker** | 914,755 (95.86) | 957,278 (34.15) | 942,368 (94.87) | 873,868 (93.32) |  |
| **Ex-smoker** | 19,175  (2.01) | 17,643  (1.76) | 17,210  (1.73) | 17,168 (1.83) |  |
| **Current smoker** | 20,349  (2.13) | 27,270  (2.72) | 33,784  (3.4) | 45,383  (4.85) |  |
| **Low income (%)** | 306,032 (32.07) | 326,224 (32.55) | 323,846 (32.60) | 306,544 (32.74) | <.0001 |
| **Drinker (%)** | 232,173 (24.33) | 258,574 (25.8) | 252,416 (25.41) | 242,997 (25.95) | <.0001 |
| **Hypertension (%)** | 120,181 (12.59) | 183,549 (18.31) | 263,851 (26.56) | 347,654 (37.13) | <.0001 |
| **Dyslipidemia (%)** | 87,680  (9.19) | 143,446 (14.31) | 217,774 (21.92) | 304,583 (32.53) | <.0001 |
| **Metabolic syndrome (%)** | 107,351 (11.25) | 182,292  (18.19) | 293,414 (29.54) | 428,744 (45.79) | <.0001 |
| **Fasting glucose (mg/dl)*** | 90.51 ± 13.86 | 92.26 ± 16.14 | 95.29 ± 19.95 | 101.26 ± 27.03 | <.0001 |

**S2 Table. Baseline characteristic by serum GGT level in women (*N* = 3,886,251)**

GGT, gamma-glutamyltransferase.

*Value was presented with mean ± SD.
