# Supplementary material for: Risk of gastrointestinal cancer in patients with an elevated level of gamma-glutamyltransferase: A nationwide population-based study
Source: PLoS One. 2021 Feb 5;16(2):e0245052. doi: 10.1371/journal.pone.0245052 (PMC7864398; doi:10.1371/journal.pone.0245052)

PLOS ONE

Risk of Gastrointestinal Cancer in Patients with an Elevated Level of Gamma-glutamyltransferase: A Nationwide Population-Based Study.

--Manuscript Draft--

**Manuscript Number:**	PONE-D-20-12359
**Article Type:**	Research Article
**Full Title:**	Risk of Gastrointestinal Cancer in Patients with an Elevated Level of Gamma-glutamyltransferase: A Nationwide Population-Based Study.
**Short Title:**	Association between elevated GGT level and risk of gastrointestinal cancer
**Corresponding Author:**	Hyun Jung Lee<br>Seoul National University College of Medicine<br>Seoul, KOREA, REPUBLIC OF
**Keywords:**	gamma-Glutamyltransferase; Hyperglycemia; Gastrointestinal Neoplasms; Cohort Studies; Stomach Neoplasms; Colorectal Neoplasms.
**Abstract:**	Emerging evidence that an elevated serum gamma-glutamyltransferase (GGT) level is associated with an increased risk of gastrointestinal cancer, but still controversial. The aim of this study to assess the relationship between GGT level and risk of gastrointestinal cancer, and the contribution of the interaction of hyperglycemia with elevated GGT level to the incidence of gastrointestinal cancer by the stratified analysis. A total of 8,120,665 Koreans who received medical checkups in 2009 were included. Subjects were classified according to the quartile of GGT level for women and men. The incidence rates of gastrointestinal cancer for each group were analyzed using Cox proportional hazards models. During follow-up, 129,853 cases of gastrointestinal cancer newly occurred (esophagus, 3,792; stomach, 57,932; and colorectal, 68,789 cases). The highest GGT quartile group showed an increased risk of gastrointestinal cancer (esophagus, hazard ratio = 2.408 [95% confidence interval, 2.184–2.654]; stomach, 1.121 [1.093–1.149]; and colorectal, 1.185 [1.158–1.211]). The risk increased significantly with the rise in GGT quartile level, regardless of the site of cancer. The stratified analysis according to glycemic status showed that the effect of elevated GGT was predominant in the risk of esophageal cancer. The effect of elevated GGT further increased the risk of stomach and colorectal cancers in diabetic patients. An elevated level of GGT was associated with an increased risk of gastrointestinal cancer, regardless of the site of cancer. The effect of the increase in GGT level on the risk of gastrointestinal cancer depended on the type of cancer and glycemic status.
**Order of Authors:**	Seung Wook Hong
	Hyun Jung Lee
	Kyungdo Han
	Jung Min Moon
	Seona Park
	Hosim Soh
	Eun Ae Kang
	Jaeyoung Chun
	Jong Pil Im
	Joo Sung Kim
**Additional Information:**	
**Question**	**Response**
**Financial Disclosure**<br><br>Enter a financial disclosure statement that	The author(s) received no specific funding for this work.

Powered by Editorial Manager® and ProduXion Manager® from Aries Systems Corporation

describes the sources of funding for the work included in this submission. Review the submission guidelines for detailed requirements. View published research articles from *PLOS ONE* for specific examples.<br><br>This statement is required for submission and **will appear in the published article** if the submission is accepted. Please make sure it is accurate.<br><br>**Unfunded studies**<br>Enter: *The author(s) received no specific funding for this work.*<br><br>**Funded studies**<br>Enter a statement with the following details:<br>• Initials of the authors who received each award<br>• Grant numbers awarded to each author<br>• The full name of each funder<br>• URL of each funder website<br>• Did the sponsors or funders play any role in the study design, data collection and analysis, decision to publish, or preparation of the manuscript?<br>• **NO** - Include this sentence at the end of your statement: *The funders had no role in study design, data collection and analysis, decision to publish, or preparation of the manuscript.*<br>• **YES** - Specify the role(s) played.<br><br>* typeset	
**Competing Interests**<br><br>Use the instructions below to enter a competing interest statement for this submission. On behalf of all authors, disclose any competing interests that could be perceived to bias this work—acknowledging all financial support and any other relevant financial or non-financial competing interests.<br><br>This statement **will appear in the published article** if the submission is accepted. Please make sure it is accurate. View published research articles from *PLOS ONE* for specific examples.	The authors have declared that no competing interests exist.

Powered by Editorial Manager® and ProduXion Manager® from Aries Systems Corporation

**NO authors have competing interests**<br><br>Enter: *The authors have declared that no competing interests exist.*<br><br>**Authors with competing interests**<br><br>Enter competing interest details beginning with this statement:<br><br>*I have read the journal's policy and the authors of this manuscript have the following competing interests: [insert competing interests here]*<br><br>* typeset	
**Ethics Statement**<br><br>Enter an ethics statement for this submission. This statement is required if the study involved:<br><br>• Human participants<br>• Human specimens or tissue<br>• Vertebrate animals or cephalopods<br>• Vertebrate embryos or tissues<br>• Field research<br><br>Write "N/A" if the submission does not require an ethics statement.<br><br>General guidance is provided below. Consult the submission guidelines for detailed instructions. **Make sure that all information entered here is included in the Methods section of the manuscript.**	This study was approved by the Institutional Review Board at Seoul National University Hospital, and the requirement for informed consent was waived since the data were analyzed anonymously (IRB approved data: 2019.06.07, No. of IRB: E-1906-008-1036).

Powered by Editorial Manager® and ProduXion Manager® from Aries Systems Corporation

**Format for specific study types**<br><br>**Human Subject Research (involving human participants and/or tissue)**<br>• Give the name of the institutional review board or ethics committee that approved the study<br>• Include the approval number and/or a statement indicating approval of this research<br>• Indicate the form of consent obtained (written/oral) or the reason that consent was not obtained (e.g. the data were analyzed anonymously)<br><br>**Animal Research (involving vertebrate animals, embryos or tissues)**<br>• Provide the name of the Institutional Animal Care and Use Committee (IACUC) or other relevant ethics board that reviewed the study protocol, and indicate whether they approved this research or granted a formal waiver of ethical approval<br>• Include an approval number if one was obtained<br>• If the study involved *non-human primates*, add *additional details* about animal welfare and steps taken to ameliorate suffering<br>• If anesthesia, euthanasia, or any kind of animal sacrifice is part of the study, include briefly which substances and/or methods were applied<br><br>**Field Research**<br><br>Include the following details if this study involves the collection of plant, animal, or other materials from a natural setting:<br>• Field permit number<br>• Name of the institution or relevant body that granted permission	
**Data Availability**<br><br>Authors are required to make all data underlying the findings described fully available, without restriction, and from the time of publication. PLOS allows rare exceptions to address legal and ethical concerns. See the PLOS Data Policy and FAQ for detailed information.	No - some restrictions will apply

A Data Availability Statement describing where the data can be found is required at submission. Your answers to this question constitute the Data Availability Statement and **will be published in the article**, if accepted.<br><br>**Important:** Stating 'data available on request from the author' is not sufficient. If your data are only available upon request, select 'No' for the first question and explain your exceptional situation in the text box.<br><br>Do the authors confirm that all data underlying the findings described in their manuscript are fully available without restriction?	
**Describe where the data may be found in full sentences. If you are copying our sample text, replace any instances of XXX with the appropriate details.**<br><br>• If the data are **held or will be held in a public repository**, include URLs, accession numbers or DOIs. If this information will only be available after acceptance, indicate this by ticking the box below. For example: *All XXX files are available from the XXX database (accession number(s) XXX, XXX.).*<br>• If the data are all contained **within the manuscript and/or Supporting Information files**, enter the following: *All relevant data are within the manuscript and its Supporting Information files.*<br>• If neither of these applies but you are able to provide **details of access elsewhere**, with or without limitations, please do so. For example:<br><br>*Data cannot be shared publicly because of [XXX]. Data are available from the XXX Institutional Data Access / Ethics Committee (contact via XXX) for researchers who meet the criteria for access to confidential data.*<br><br>*The data underlying the results presented in the study are available from (include the name of the third party*	Data cannot be shared officially because this study was conducted using the data from the claim database of the National Health Insurance Service in Korea. Data are available from the National Health Insurance Service Institutional Data Access / Ethics Committee for researchers who meet the criteria for access to confidential data.

and contact information or URL).<br>• This text is appropriate if the data are owned by a third party and authors do not have permission to share the data.<br><br>* typeset	
Additional data availability information:	

Manuscript
Click here to download Manuscript Manuscript_20200427_PLOSONE_Plain.docx

Title: Risk of Gastrointestinal Cancer in Patients with an Elevated Level of Gamma-glutamyltransferase: A Nationwide Population-Based Study.

Short title: Association between elevated GGT level and risk of gastrointestinal cancer

Seung Wook Hong[1,#a], Hyun Jung Lee[1*], Kyungdo Han[2], Jung Min Moon[1], Seona Park[1], Hosim Soh[1], Eun Ae Kang[1], Jaeyoung Chun[3], Jong Pil Im[1], and Joo Sung Kim[1].

[1]Department of Internal Medicine and Liver Research Institute, Seoul National University College of Medicine, Seoul, Korea

[2]Department of Medical Statistics, The Catholic University of Korea College of Medicine, Seoul, Korea

[3]Department of Internal Medicine, Gangnam Severance Hospital, Yonsei University College of Medicine, Seoul, Korea

[#a]Current Address: Department of Gastroenterology, Asan Medical Center, University of Ulsan College of Medicine, Seoul, South Korea

[*]**Corresponding author:** Hyun Jung Lee, MD, PhD.

Associate professor of Internal Medicine,

Department of Internal Medicine and Liver Research Institute,

Seoul National University College of Medicine.

101 Daehak-ro, Jongno-gu, Seoul 03080, Korea
Tel: +82-2-2072-2228, Fax: +82-2-740-8112, E-mail: guswjd80@gmail.com

Abstract

Emerging evidence that an elevated serum gamma-glutamyltransferase (GGT) level is associated with an increased risk of gastrointestinal cancer, but still controversial. The aim of this study to assess the relationship between GGT level and risk of gastrointestinal cancer, and the contribution of the interaction of hyperglycemia with elevated GGT level to the incidence of gastrointestinal cancer by the stratified analysis. A total of 8,120,665 Koreans who received medical checkups in 2009 were included. Subjects were classified according to the quartile of GGT level for women and men. The incidence rates of gastrointestinal cancer for each group were analyzed using Cox proportional hazards models. During follow-up, 129,853 cases of gastrointestinal cancer newly occurred (esophagus, 3,792; stomach, 57,932; and colorectal, 68,789 cases). The highest GGT quartile group showed an increased risk of gastrointestinal cancer (esophagus, hazard ratio = 2.408 [95% confidence interval, 2.184–2.654]; stomach, 1.121 [1.093–1.149]; and colorectal, 1.185 [1.158–1.211]). The risk increased significantly with the rise in GGT quartile level, regardless of the site of cancer. The stratified analysis according to glycemic status showed that the effect of elevated GGT was predominant in the risk of esophageal cancer. The effect of elevated GGT further increased the risk of stomach and colorectal cancers in diabetic patients. An elevated level of GGT was associated with an increased risk of gastrointestinal cancer, regardless of the site of cancer. The effect of the increase in GGT level on the risk of gastrointestinal cancer depended on the type of cancer and glycemic status.

Introduction

Serum gamma-glutamyltransferase (GGT) is an indicator related to hepatic dysfunction and used as a surrogate marker that reflects excessive alcohol consumption [1, 2]. Several epidemiologic studies have reported that elevated GGT level is associated with the incidence of various disease including metabolic syndrome, diabetes mellitus (DM), and cardiovascular disease [3-8]. Emerging evidence indicates that an elevated GGT level is also linked to increased risk of cancer. Notably, many studies have demonstrated a positive correlation between an elevated GGT level and the risk of gastrointestinal cancer, but results have been inconsistent depending on sex and the site of cancer [9-14]. Hence, further study with a large population is needed to establish any association between elevated GGT level and risk of gastrointestinal cancer.

Diabetes mellitus is a chronic disease of increasing prevalence, generally accompanied by hyperglycemia caused by insulin resistance [15]. An elevated GGT level is related to hyperglycemia, and many studies have reported that patients with DM have an increased risk of gastrointestinal cancer [16-22]. However, few studies have assessed the association of serum GGT level and glycemic status with the incidence of gastrointestinal cancer in the general population, and little is known about whether the effect of GGT on the gastrointestinal cancer risk is influenced by glycemic status.

Therefore, we aimed to assess whether an elevated serum GGT level is associated with the risk of gastrointestinal cancer through a large-scale study using nationwide

data. In addition, we evaluated the effect of glycemic status on the association between an elevated GGT level and the risk of gastrointestinal cancer, and the contribution of the interaction between these two factors on the incidence of gastrointestinal cancer.

Materials and methods

Data sources

This population-based study was retrospectively conducted using the database from the National Health Insurance Service (NHIS) in South Korea. The NHIS is one of the social security systems provided by the government, and all Koreans are obliged to enroll in. It serves to pay health care providers for medical services on behalf of the general population. All data related to the medical services guaranteed by the NHIS are stored in this database. The NHIS database collects demographic data as well as the information regarding medical services, including medications, hospitalization, and diagnoses identified by the International Classification of Disease, Tenth Revision (ICD-10) codes of the subscribers. The NHIS recommends that subscribers undergo a general medical check-up biennially. This general medical check-up includes body measurements, chest X-ray, and blood chemistry tests.

Data collection and study population

Demographic information including age, sex, and income was stored in the NHIS database for all participants who underwent general medical checkups. The weight, height, and blood pressure for participants were measured, and laboratory tests including fasting blood glucose (FBG), aspartate aminotransferase, alanine aminotransferase, GGT level, and cholesterol were performed on the same day. A self-reporting questionnaire was used to assess drinking habits, history of smoking, income, and exercise habits for the participants. The history of smoking was classified into three categories: (1) non-smoker; (2) ex-smoker; and (3) current smoker. An ex-smoker was defined as a person with smoking experience of >100 cigarettes, although smoking had currently ceased. A heavy drinker was defined as a person drinking >30 g of alcohol per week.

Adults over the age of 20 years who underwent a general medical check-up provided by the NHIS during January–December 2009 qualified for registration in this cohort. At the time of enrolment, patients who were previously diagnosed with cancer identified by ICD-10, regardless of the type, were excluded. To minimize disruption of pre-existing factors to the incidence of gastrointestinal cancer, any type of cancer patients who were diagnosed within one year of the time of enrolment were excluded from the cohort. Individuals diagnosed with liver cirrhosis (K703) or hepatitis (K746) were also excluded. To avoid the confounding effect of excessive alcohol consumption on the serum GGT level, individuals with heavy drinking were excluded from the cohort.

Outcomes and ethical considerations

Subjects in the cohort were divided into four groups according to the quartile level of GGT. The quartile level of GGT was differently determined, considering differences in the level of GGT by sex (men: first quartile [Q1], <21 U/L; second quartile [Q2], 21–29 U/L; third quartile [Q3], 30–47 U/L; and fourth quartile [Q4], ≥48 U/L; and women: Q1, <13 U/L; Q2, 13–15 U/L; Q3, 16–22 U/L; and Q4, ≥23 IU/L). The primary outcome was to evaluate the incidence rate of gastrointestinal cancer for each group by the quartile level of GGT. The secondary outcome was indirectly to assess the interaction of an elevated GGT level and hyperglycemia contributing to the risk of gastrointestinal cancer, using stratified analysis according to glycemic status. The glycemic status was categorized in accordance with FBG (non-DM, FBG < 100 mg/dL; impaired fasting glucose (IFG), 100 ≤ FBG < 126 mg/dL; and DM, FBG ≥ 126 mg/dL). The follow-up was terminated at the occurrence of gastrointestinal cancer and monitoring of other members of the cohort was continued to until 31 December 2017. The gastrointestinal cancer included esophageal, stomach, and colorectal cancers. Cases of newly diagnosed gastrointestinal cancer during the follow-up period were identified using ICD-10 codes in the NHIS database as follows – esophageal (C15), stomach (C16), and colorectal cancer (C18–20) – and their index date was the first day that cancer was recorded. This study was approved by the Institutional Review Board at Seoul National University Hospital, and the requirement for informed consent was waived (IRB approved data: 2019.06.07, No. of IRB: E-1906-008-1036).

Statistical analysis

Data were expressed as mean ± standard deviation for continuous variables and as number and proportions for categorical variables. To compare differences among groups, Student's t-test and ANOVA were used for continuous data and the chi-square test for categorical data. The incidence rate of gastrointestinal cancer was calculated by dividing the number of incident cases by the overall follow-up period and presented as 10,000 person-years. The Cox proportional hazard model was used to evaluate the association GGT level and risk of gastrointestinal cancer. In addition, stratified analyses were conducted according to glycemic status. Two adjustment models were applied, model 1 was adjusted for age and sex, and model 2 was adjusted for age, sex, BMI, smoking, drinking, exercise, and income. A statistical significance was defined as $P < 0.05$. Statistical analyses were performed using SAS version 9.4 (SAS Institute, Cary, NC, USA).

Results

Baseline characteristics

A total of 8,120,665 individuals were enrolled in this cohort. The selection process for the study subjects is illustrated in Fig 1. The eligible population was categorized into four groups by the quartile level of serum GGT. The baseline characteristics of the population in accordance with GGT quartiles are presented in Table 1. Compared with the lower GGT quartile groups (Q1–3), more individuals who drank or smoked were included in the higher GGT quartile group (Q4). The proportion of subjects with hypertension, dyslipidemia, and metabolic syndrome gradually increased as the GGT

quartile level increased. The level of FBG also increased as the level of GGT quartile increased.

Fig 1. **Flow chart of selection process of study subject**

Table1. Baseline characteristics by serum GGT level (*N* = 8,120,665)

	**GGT quartile**				***P*-value**
	**Q1**	**Q2**	**Q3**	**Q4**	
	***N* = 2,001,551**	***N* = 2,029,189**	***N* = 2,099,258**	***N* = 1,990,667**	
**Age (years)***	43.75 ± 14.75	45.93 ± 14.36	47.87 ± 13.83	49.28 ± 12.9	<.0001
**Sex, Men (%)**	1,047,272 (52.32)	1,026,998 (50.61)	1,105,896 (52.68)	1,054,248 (52.96)	<.0001
**Waist circumference (cm)***	76.34 ± 8.1	78.36 ± 8.66	80.92 ± 8.91	83.5 ± 8.88	<.0001
**Body mass index (kg/m²)***	22.37 ± 2.71	23.1 ± 2.96	24.02 ± 3.16	24.97 ± 3.34	<.0001
**Exercise (%)**	1,036,622 (51.79)	1,044,128 (51.46)	1,074,483 (51.18)	1,000,684 (50.27)	<.0001
**Smoking status (%)**					<.0001
**Non-smoker**	1,330,105 (66.45)	1,308,008 (64.46)	1,272,410 (60.61)	1,140,435 (57.29)	
**Ex-smoker**	259,333 (12.96)	269,649 (13.29)	294,621 (14.03)	261,707 (13.15)	
**Current smoker**	412,113 (20.59)	451,532 (22.25)	532,227 (25.35)	588,525 (29.56)	
**Low income (%)**	425,064 (21.24)	429,320 (21.16)	440,156 (20.97)	425,771 (21.39)	<.0001
**Drinker (%)**	783,461 (39.14)	876,698 (43.2)	997,037 (47.49)	1,060,510 (53.27)	<.0001
**Hypertension (%)**	290,349 (14.51)	403,676 (19.89)	564,871 (26.91)	713,722 (35.85)	<.0001
**Dyslipidemia (%)**	165,360 (8.26)	272,186 (13.41)	420,969 (20.05)	577,006 (28.99)	<.0001
**Metabolic syndrome (%)**	218,539 (10.92)	371,250 (18.3)	607,707 (28.95)	869,756 (43.69)	<.0001
**Fasting glucose (mg/dl)***	92.13 ± 16.49	94.07 ± 18.82	97.15 ± 22.32	102.55 ± 27.88	<.0001

GGT, gamma-glutamyltransferase.

*Value was presented with mean ± SD.

Association between serum GGT level and the risk of gastrointestinal cancer

During the 8-year follow-up, 3792 cases of esophageal cancer, 57,932 cases of stomach cancer, and 68,789 cases of colorectal cancer developed. For each type of gastrointestinal cancer, the number of incident cases, the incidence rate, and the adjusted hazard ratio (HR) according to the GGT quartile level are shown in Table 2. After adjusting for age, sex, BMI, smoking, drinking, exercise, and income, the risk of gastrointestinal cancer significantly increased with a gradual rise of the serum GGT level, regardless of the type of cancer. The adjusted HRs for esophageal cancer incidence for Q2, Q3, and Q4 in comparison with Q1 were 1.218 [95% confidence interval (95%CI), 1.098–1.351], 1.445 [1.305–1.559], and 2.408 [2.184–2.654], respectively (*p* for trend <0.001). The adjusted HRs for stomach cancer incident for Q2, Q3, and Q4 in comparison with Q1 were 1.012 [0.987–1.037], 1.042 [1.017–1.068], and 1.121 [1.093–1.149], respectively (*p* for trend <0.001). Similarly, the adjusted HRs for colorectal cancer incidence for Q2, Q3, and Q4 in comparison with Q1 were 1.030 [1.007–1.054], 1.091 [1.067–1.116], and 1.185 [1.158–1.212], respectively (*p* for trend <0.001) (Fig 2).

Fig 2. Association between serum GGT quartile level and risk of gastrointestinal cancer

(a) esophageal cancer, (b) stomach cancer, and (c) colorectal cancer. Q1-4: GGT quartile level

Table 2. Risk of gastrointestinal cancer according to serum GGT quartile level

		GGT quartile				***P* for trend**
**Cancer site**		**Q1**	**Q2**	**Q3**	**Q4**	
**Esophagus**	Cases, n	671	780	945	1,396	
	Incidence rate[1]	0.46	0.53	0.62	0.97	
	Model 1, HR (95% CI)[2]	1*	1.194 (1.077-1.324)	1.403 (1.270-1.549)	2.415 (2.200-2.651)	<0.001
	Model 2, HR (95% CI)[3]	1*	1.218 (1.098-1.351)	1.445 (1.305-1.559)	2.408 (2.184-2.654)	<0.001
**Stomach**	Cases, n	12,406	13,530	15,470	15,886	
	Incidence rate[1]	8.52	9.17	10.15	11.04	
	Model 1, HR (95% CI)[2]	1*	1.028 (1.003-1.053)	1.073 (1.048-1.099)	1.172 (1.144-1.200)	<0.001
	Model 2, HR (95% CI)[3]	1*	1.012 (0.987-1.037)	1.042 (1.017-1.068)	1.121 (1.093-1.149)	<0.001
**Colorectal**	Cases, n	13,749	15,802	18,953	20,265	
	Incidence rate[1]	9.45	10.72	12.45	14.09	
	Model 1, HR (95% CI)[2]	1*	0.983 (0.918-1.052)	1.075 (1.010-1.145)	1.209 (1.139-1.284)	<0.001
	Model 2, HR (95% CI)[3]	1*	1.03 (1.007-1.054)	1.091 (1.067-1.116)	1.185 (1.158-1.212)	<0.001

GGT, gamma-glutamyltransferase.

*Reference value

[1]Per 10,000 person-year, [2]Adjusted for age, sex,[3]Adjusted for age, sex, BMI, smoking, drinking, exercise, income.

Association between hyperglycemia and risk of gastrointestinal cancer

For each type of gastrointestinal cancer, the number of incident cases and incidence rates stratified by glycemic status are presented in Table 3. After multivariable adjustment, compared to the non-DM group, the incidence of each gastrointestinal cancer was higher in both IFG and DM group (esophagus, HR: 1.198 [1.113–1.290] in

the IFG group and 1.278 [1.170–1.397] in the DM group; stomach: 1.050 [1.030–1.071] in the IFG group and 1.181 [1.154–1.210] in the DM group; and colorectal: 1.089 [1.069–1.108] in the IFG group and 1.217 [1.191–1.244] in the DM group).

Table 3. Risk of gastrointestinal cancer according to glycemic status

		Glycemic status		
**Cancer site**		**Non-DM**	**IFG**	**DM**
**Esophagus**	Cases, n	1,996	1,122	674
	Incidence rate[1]	0.48	0.87	1.44
	Model 1, HR (95% CI)[2]	1*	1.155 (1.074-1.243)	1.168 (1.070-1.276)
	Model 2, HR (95% CI)[3]	1*	1.198 (1.113-1.290)	1.278 (1.170-1.397)
**Stomach**	Cases, n	32,862	18,214	10,951
	Incidence rate[1]	7.93	11.74	20.03
	Model 1, HR (95% CI)[2]	1*	1.056 (1.036-1.077)	1.191 (1.164-1.220)
	Model 2, HR (95% CI)[3]	1*	1.050 (1.030-1.071)	1.181 (1.154-1.210)
**Colorectal**	Cases, n	39,604	18,214	10,951
	Incidence rate[1]	9.57	14.14	23.60
	Model 1, HR (95% CI)[2]	1*	1.109 (1.089-1.128)	1.246 (1.220-1.274)
	Model 2, HR (95% CI)[3]	1*	1.089 (1.069-1.108)	1.217 (1.191-1.244)

DM, Diabetes mellitus; IFG: Impaired fasting glucose.

*Reference value

[1]Per 10,000 person-year, [2]Adjusted for age, sex,[3]Adjusted for age, sex, BMI, smoking, drinking, exercise, income.

Effect of serum GGT level on the risk of gastrointestinal cancer stratified by glycemic status

Stratified analyses by glycemic status were conducted to evaluate the interaction of an elevated GGT level and hyperglycemia contributing to the risk of gastrointestinal

cancer. The subjects were stratified into non-DM, IFG, and DM groups by glycemic status, and each group was divided into Q4 groups and other groups (Q1–3) in accordance with the serum GGT quartile level. After setting Q1–3 groups in non-DM as a reference group, the adjusted HR of the incidence in gastrointestinal cancer was estimated for each group (Table 4).

An elevated GGT level had a significant effect on the incidence of esophageal cancer. The risk of esophageal cancer was higher in the Q4 than Q1–3 groups regardless of glycemic status. Additionally, the Q4 groups of non-DM and IFG showed a higher risk of esophageal cancer than the Q1–3 group of DM (non-DM/Q4, HR: 1.98 [95%CI, 1.799–2.180]; IFG/Q4, 2.089 [1.873–2.331]; and DM/Q1-3, 1.168 [1.037–1.316]) (Fig 3a).

An elevated serum GGT level and hyperglycemia showed an interactive effect on the incidence of stomach and colorectal cancer. For stomach cancer, the Q4 group of DM showed the highest risk (HR: 1.283 [95%CI: 1.237–1.331]), and the HR did not differ between the Q4 group of IFG and the Q1–3 group of DM (IFG/Q4, HR: 1.129 [95%CI: 1.094–1.165]; and DM/Q1-3, 1.164 [1.130–1.199]) (Fig 3b).

The risk of colorectal cancer was highest in the Q4 group of DM (HR: 1.342 [95%CI: 1.299–1.386]). In contrast, the HR in the Q4 group of IFG was higher than that in the Q1–3 group of DM, although not significant (IFG/Q4, HR: 1.214 [95%CI: 1.180–1.248]; and DM/Q1-3, 1.193 [1.161–1.227]) (Fig 3c).

Fig 3. Effect of an elevated GGT level on the risk of gastrointestinal cancer stratified by glycemic status

(a) esophageal cancer, (b) stomach cancer, and (c) colorectal cancer. DM: diabetes mellitus IFG: impaired fasting glucose, Q1-4: GGT quartile level.

Table 4. Association risk of gastrointestinal cancer and serum GGT level stratified by glycemic status

Glycemic status	GGT level	Cancer site					
		Esophagus		Stomach		Colorectal	
		Incidence rate[1]	HR (95% CI)[2]	Incidence rate[1]	HR (95% CI)[2]	Incidence rate[1]	HR (95% CI)[2]
**Non-DM**	Q1-Q3	0.41	1*	7.68	1*	9.11	1*
	Q4	0.76	1.98 (1.799-2.180)	9.02	1.077 (1.049-1.105)	11.44	1.093 (1.068-1.119)
**IFG**	Q1-Q3	0.77	1.193 (1.088-1.309)	11.71	1.043 (1.019-1.067)	13.61	1.067 (1.044-1.090)
	Q4	1.09	2.089 (1.873-2.331)	11.90	1.129 (1.094-1.165)	15.41	1.214 (1.180-1.248)
**DM**	Q1-Q3	1.32	1.168 (1.037-1.316)	21.51	1.164 (1.130-1.199)	24.17	1.193 (1.161-1.227)
	Q4	1.60	2.341 (2.063-2.656)	18.01	1.283 (1.237-1.331)	22.85	1.342 (1.299-1.386)

DM: Diabetes Mellitus, IFG: Impaired fasting glucose.

*Reference value

[1]Per 10,000 person-year, [2]Adjusted for age, sex, BMI, smoking, drinking, exercise, income.

Discussion

In this nationwide population-based study, we demonstrated that an elevated serum GGT level was significantly associated with the risk of gastrointestinal cancer. In regard to site-specific cancer incidence, the risks of esophageal, stomach, and colorectal cancers all increased as the GGT level increased. Moreover, the risk of gastrointestinal cancer was also related to hyperglycemia including IFG. The stratified analysis of the association between an elevated GGT level and the risk of gastrointestinal cancer by glycemic status showed that an elevated GGT level had a

dominant effect on incidence of esophageal cancer. Notably, an elevated GGT level and hyperglycemia showed an interactive effect on the incidence of stomach and colorectal cancers. To the best of our knowledge, this is the first large-scale study to confirm the association of elevated GGT level and hyperglycemia with the risk of gastrointestinal cancer and to reveal the interaction of the two factors.

Our findings are consistent with previous several epidemiologic studies reporting a significant correlation between an elevated GGT level and incidence of gastrointestinal cancer. Cohort studies from Austria and Sweden revealed that subjects with high serum GGT levels had an increased risk of gastrointestinal cancer [9-11], and a meta-analysis found a positive association between serum GGT levels and the risk of gastrointestinal cancer [13]. Another study in Korea showed that an elevated GGT level was associated with an increased risk of esophageal, stomach, and colorectal cancers, but this was significant only in men [14]. Although the underlying mechanism for the link between an elevated GGT level and the risk of gastrointestinal cancer is not completely understood, an elevated serum GGT level may increase the risk of cancer via the oxidative stress pathway related to glutathione metabolism [23]. Several preclinical studies identified that increased GGT activity can be a response to oxidative stress and, in turn, the persistent production of reactive oxygen species by increased GGT activity may cause genetic instability and tumor progression [23-27]. These findings are the most plausible mechanisms related to the association between an elevated GGT level and increased risk of gastrointestinal cancer.

Although there was a difference in degree for the specific site of cancer, an elevated

GGT level and hyperglycemia were both related to the increased risk of gastrointestinal cancer and the risk was highest in the high GGT group of DM patients. Moreover, it is well known that an elevated GGT level is associated with hyperglycemia because several studies found that an elevated GGT level may be involved in the development of prediabetes or diabetes [28-30]. There is corroborative evidence to support the correlations between pairs among an elevated GGT level, hyperglycemia, and carcinogenesis. Insulin resistance is a pivotal mechanism to develop prediabetes and type 2 DM, and it causes hyperinsulinemia [15]. Although it is still uncertain, hyperinsulinemia may promote cell proliferation via various pathways and ultimately contribute to the incidence of cancer [31-33]. In addition, increased oxidative stress may induce insulin resistance and pancreatic β-cell dysfunction [34, 35]. Chronic inflammation is closely related to both insulin resistance and increased oxidative stress and has been recognized as a critical factor for carcinogenesis [36, 37]. It is presumed that a series of these factors interact with one another, affecting the development of cancer. Further studies are needed to investigate the cause of differences in the interaction between an elevated GGT level and hyperglycemia according to the type of cancer, and the fundamental mechanism of carcinogenesis.

It is remarkable that the impact of an elevated GGT level and hyperglycemia on the incidence of gastrointestinal cancer differed with the specific site of cancer. The effect of high GGT level was overwhelming in developing esophageal cancer. However, the interactive effect of an elevated GGT level and hyperglycemia, which contributed to the risk of stomach and colorectal cancer, was indirectly revealed. Considering the higher tendency for colorectal cancer risk in patients with high GGT level of the IFG

group compared with a low GGT level of the DM group, the serum GGT level has potential as a discriminating marker in predicting colorectal cancer, especially in prediabetic patients. Therefore, development of a model is required to precisely estimate an individual’s risk of developing gastrointestinal cancer based on serum GGT level and glycemic status.

This was a large-scale cohort study with nearly 8.1 million participants. However, this study had several limitations. First, it was limited to assessing the effect of lowering the GGT level or FBG on the incidence of gastrointestinal cancer, because only the baseline levels were collected. Further longitudinal studies are needed to evaluate the change in the incidence of gastrointestinal cancer as the level of GGT changes. In addition, the definition of DM in this study did not include all the diagnostic criteria for DM. Although this definition has been widely used in previous nationwide studies [38], additional studies with a more reliable definition for DM are required. To dispel concern due to the confounding effect of hepatic dysfunction on the outcomes, we excluded patients with liver cirrhosis or hepatitis. However, the results for liver function data including of liver enzymes were not adjusted. Finally, the results on the interaction between an elevated GGT level and glycemic status should be interpreted cautiously because they were estimated with the indirect statistical method of stratified analysis.

In conclusion, this large-scale study showed that an elevated GGT level was associated with an increased risk of gastrointestinal cancer. Additionally, the effect of an elevated GGT level on the cancer incidence varied with the type of cancer and glycemic status. Further studies are needed to assess the effect of sequential change in GGT level and glycemic status on the incidence of gastrointestinal cancer. Despite

some limitations, this study provides meaningful evidence for clinicians to develop a strategy for screening of gastrointestinal cancer according to GGT level and glycemic status.

References

1. Teschke R, Rauen J, Neuefeind M, Petrides AS, Strohmeyer G. Alcoholic liver disease associated with increased gamma-glutamyltransferase activities in serum and liver. Adv Exp Med Biol. 1980;132:647-54. Epub 1980/01/01. doi: 10.1007/978-1-4757-1419-7_67. PubMed PMID: 6106999.

2. Whitfield JB. Gamma glutamyl transferase. Crit Rev Clin Lab Sci. 2001;38(4):263-355. Epub 2001/09/21. doi: 10.1080/20014091084227. PubMed PMID: 11563810.

3. Melvin JC, Rodrigues C, Holmberg L, Garmo H, Hammar N, Jungner I, et al. Gamma-glutamyl transferase and C-reactive protein as alternative markers of metabolic abnormalities and their associated comorbidites: a prospective cohort study. Int J Mol Epidemiol Genet. 2012;3(4):276-85. Epub 2012/12/04. PubMed PMID: 23205179; PubMed Central PMCID: PMCPMC3508539.

4. Kunutsor SK, Abbasi A, Adler AI. Gamma-glutamyl transferase and risk of type II diabetes: an updated systematic review and dose-response meta-analysis. Ann Epidemiol. 2014;24(11):809-16. Epub 2014/09/30. doi: 10.1016/j.annepidem.2014.09.001. PubMed PMID: 25263236.

5. Franzini M, Scataglini I, Ricchiuti A, Fierabracci V, Paolicchi A, Pompella A, et al. Association between plasma gamma-glutamyltransferase fractions and metabolic syndrome among hypertensive patients. Sci Rep. 2017;7(1):12003. Epub 2017/09/22. doi: 10.1038/s41598-017-12356-w. PubMed PMID: 28931896; PubMed Central PMCID: PMCPMC5607275.

6. Nano J, Muka T, Ligthart S, Hofman A, Darwish Murad S, Janssen HLA, et al. Gamma-glutamyltransferase levels, prediabetes and type 2 diabetes: a Mendelian randomization study. Int J Epidemiol. 2017;46(5):1400-9. Epub 2017/03/25. doi: 10.1093/ije/dyx006. PubMed PMID: 28338987.

7. Wang S, Zhang J, Zhu L, Song L, Meng Z, Jia Q, et al. Association between liver function and metabolic syndrome in Chinese men and women. Sci Rep. 2017;7:44844. Epub 2017/03/21. doi: 10.1038/srep44844. PubMed PMID: 28317840; PubMed Central PMCID: PMCPMC5357848.

8. Ndrepepa G, Colleran R, Kastrati A. Gamma-glutamyl transferase and the risk of atherosclerosis and coronary heart disease. Clin Chim Acta. 2018;476:130-8. Epub 2017/11/28. doi: 10.1016/j.cca.2017.11.026. PubMed PMID: 29175647.

9. Strasak AM, Pfeiffer RM, Klenk J, Hilbe W, Oberaigner W, Gregory M, et al. Prospective study of the association of gamma-glutamyltransferase with cancer incidence in women. Int J Cancer. 2008;123(8):1902-6. Epub 2008/08/09. doi: 10.1002/ijc.23714. PubMed PMID: 18688855.

10. Strasak AM, Rapp K, Brant LJ, Hilbe W, Gregory M, Oberaigner W, et al. Association of gamma-glutamyltransferase and risk of cancer incidence in men: a prospective study. Cancer Res. 2008;68(10):3970-7. Epub 2008/05/17. doi: 10.1158/0008-5472.Can-07-6686. PubMed PMID: 18483283; PubMed Central

PMCID: PMCPMC5955388.

11. Van Hemelrijck M, Jassem W, Walldius G, Fentiman IS, Hammar N, Lambe M, et al. Gamma-glutamyltransferase and risk of cancer in a cohort of 545,460 persons - the Swedish AMORIS study. Eur J Cancer. 2011;47(13):2033-41. Epub 2011/04/14. doi: 10.1016/j.ejca.2011.03.010. PubMed PMID: 21486691.

12. Tsuboya T, Kuriyama S, Nagai M, Hozawa A, Sugawara Y, Tomata Y, et al. Gamma-glutamyltransferase and cancer incidence: the Ohsaki cohort study. J Epidemiol. 2012;22(2):144-50. Epub 2012/01/27. doi: 10.2188/jea.je20110071. PubMed PMID: 22277791; PubMed Central PMCID: PMCPMC3798593.

13. Kunutsor SK, Apekey TA, Van Hemelrijck M, Calori G, Perseghin G. Gamma glutamyltransferase, alanine aminotransferase and risk of cancer: systematic review and meta-analysis. Int J Cancer. 2015;136(5):1162-70. Epub 2014/07/22. doi: 10.1002/ijc.29084. PubMed PMID: 25043373.

14. Mok Y, Son DK, Yun YD, Jee SH, Samet JM. gamma-Glutamyltransferase and cancer risk: The Korean cancer prevention study. Int J Cancer. 2016;138(2):311-9. Epub 2015/06/27. doi: 10.1002/ijc.29659. PubMed PMID: 26111622.

15. Skyler JS, Bakris GL, Bonifacio E, Darsow T, Eckel RH, Groop L, et al. Differentiation of Diabetes by Pathophysiology, Natural History, and Prognosis. Diabetes. 2017;66(2):241-55. Epub 2016/12/17. doi: 10.2337/db16-0806. PubMed PMID: 27980006; PubMed Central PMCID: PMCPMC5384660.

16. Inoue M, Iwasaki M, Otani T, Sasazuki S, Noda M, Tsugane S. Diabetes mellitus and the risk of cancer: results from a large-scale population-based cohort study in Japan. Arch Intern Med. 2006;166(17):1871-7. Epub 2006/09/27. doi: 10.1001/archinte.166.17.1871. PubMed PMID: 17000944.

17. Valent F. Diabetes mellitus and cancer of the digestive organs: An Italian population-based cohort study. J Diabetes Complications. 2015;29(8):1056-61. Epub 2015/08/16. doi: 10.1016/j.jdiacomp.2015.07.017. PubMed PMID: 26275864.

18. Dixon JL, Copeland LA, Zeber JE, MacCarthy AA, Reznik SI, Smythe WR, et al. Association between diabetes and esophageal cancer, independent of obesity, in the United States Veterans Affairs population. Dis Esophagus. 2016;29(7):747-51. Epub 2015/10/13. doi: 10.1111/dote.12402. PubMed PMID: 26455587.

19. Miao ZF, Xu H, Xu YY, Wang ZN, Zhao TT, Song YX, et al. Diabetes mellitus and the risk of gastric cancer: a meta-analysis of cohort studies. Oncotarget. 2017;8(27):44881-92. Epub 2017/04/19. doi: 10.18632/oncotarget.16487. PubMed PMID: 28415651; PubMed Central PMCID: PMCPMC5546528.

20. Xu B, Zhou X, Li X, Liu C, Yang C. Diabetes mellitus carries a risk of esophageal cancer: A meta-analysis. Medicine (Baltimore). 2017;96(35):e7944. Epub 2017/09/01. doi: 10.1097/md.0000000000007944. PubMed PMID: 28858123; PubMed Central PMCID: PMCPMC5585517.

21. Ma Y, Yang W, Song M, Smith-Warner SA, Yang J, Li Y, et al. Type 2 diabetes and risk of colorectal cancer in two large U.S. prospective cohorts. Br J Cancer. 2018;119(11):1436-42. Epub 2018/11/08. doi: 10.1038/s41416-018-0314-4. PubMed PMID: 30401889; PubMed Central PMCID: PMCPMC6265303.

22. Qi J, He P, Yao H, Song R, Ma C, Cao M, et al. Cancer risk among patients with type 2 diabetes: A real-world study in Shanghai, China. J Diabetes. 2019;11(11):878-83. Epub 2019/05/10. doi: 10.1111/1753-0407.12926. PubMed PMID: 31070008.

23. Hanigan MH. Gamma-glutamyl transpeptidase: redox regulation and drug

resistance. Adv Cancer Res. 2014;122:103-41. Epub 2014/06/30. doi: 10.1016/b978-0-12-420117-0.00003-7. PubMed PMID: 24974180; PubMed Central PMCID: PMCPMC4388159.

24. Corti A, Duarte TL, Giommarelli C, De Tata V, Paolicchi A, Jones GD, et al. Membrane gamma-glutamyl transferase activity promotes iron-dependent oxidative DNA damage in melanoma cells. Mutat Res. 2009;669(1-2):112-21. Epub 2009/06/10. doi: 10.1016/j.mrfmmm.2009.05.010. PubMed PMID: 19505483.

25. Komlosh A, Volohonsky G, Porat N, Tuby C, Bluvshtein E, Steinberg P, et al. gamma-Glutamyl transpeptidase and glutathione biosynthesis in non-tumorigenic and tumorigenic rat liver oval cell lines. Carcinogenesis. 2002;23(4):671-8. Epub 2002/06/22. doi: 10.1093/carcin/23.4.669. PubMed PMID: 12075625.

26. Hanigan MH, Gallagher BC, Townsend DM, Gabarra V. Gamma-glutamyl transpeptidase accelerates tumor growth and increases the resistance of tumors to cisplatin in vivo. Carcinogenesis. 1999;20(4):553-9. Epub 1999/05/01. doi: 10.1093/carcin/20.4.553. PubMed PMID: 10223181; PubMed Central PMCID: PMCPMC6522259.

27. Hanigan MH. gamma-Glutamyl transpeptidase, a glutathionase: its expression and function in carcinogenesis. Chem Biol Interact. 1998;111-112:333-42. Epub 1998/07/29. doi: 10.1016/s0009-2797(97)00170-1. PubMed PMID: 9679564.

28. Wu J, Qiu L, Yan WH, Cheng XQ, Wu W, Guo XZ, et al. Serum gamma-glutamyltransferase and uric acid levels are associated with impaired fasting glucose in adults from Inner Mongolia, China. BMC Public Health. 2013;13:294. Epub 2013/04/05. doi: 10.1186/1471-2458-13-294. PubMed PMID: 23552170; PubMed Central PMCID: PMCPMC3622625.

29. Sabanayagam C, Shankar A, Li J, Pollard C, Ducatman A. Serum gamma-glutamyl transferase level and diabetes mellitus among US adults. Eur J Epidemiol. 2009;24(7):369-73. Epub 2009/05/19. doi: 10.1007/s10654-009-9346-7. PubMed PMID: 19449164.

30. Nakanishi N, Suzuki K, Tatara K. Serum gamma-glutamyltransferase and risk of metabolic syndrome and type 2 diabetes in middle-aged Japanese men. Diabetes Care. 2004;27(6):1427-32. Epub 2004/05/27. doi: 10.2337/diacare.27.6.1427. PubMed PMID: 15161799.

31. Tsujimoto T, Kajio H, Sugiyama T. Association between hyperinsulinemia and increased risk of cancer death in nonobese and obese people: A population-based observational study. Int J Cancer. 2017;141(1):102-11. Epub 2017/04/09. doi: 10.1002/ijc.30729. PubMed PMID: 28390156; PubMed Central PMCID: PMCPMC5435954.

32. Orgel E, Mittelman SD. The links between insulin resistance, diabetes, and cancer. Curr Diab Rep. 2013;13(2):213-22. Epub 2012/12/29. doi: 10.1007/s11892-012-0356-6. PubMed PMID: 23271574; PubMed Central PMCID: PMCPMC3595327.

33. Hsu IR, Kim SP, Kabir M, Bergman RN. Metabolic syndrome, hyperinsulinemia, and cancer. Am J Clin Nutr. 2007;86(3):s867-71. Epub 2008/02/13. doi: 10.1093/ajcn/86.3.867S. PubMed PMID: 18265480.

34. Tangvarasittichai S. Oxidative stress, insulin resistance, dyslipidemia and type 2 diabetes mellitus. World J Diabetes. 2015;6(3):456-80. Epub 2015/04/22. doi: 10.4239/wjd.v6.i3.456. PubMed PMID: 25897356; PubMed Central PMCID: PMCPMC4398902.

35. Park K, Gross M, Lee DH, Holvoet P, Himes JH, Shikany JM, et al. Oxidative

stress and insulin resistance: the coronary artery risk development in young adults study. Diabetes Care. 2009;32(7):1302-7. Epub 2009/04/25. doi: 10.2337/dc09-0259. PubMed PMID: 19389821; PubMed Central PMCID: PMCPMC2699736.

36. Multhoff G, Molls M, Radons J. Chronic inflammation in cancer development. Front Immunol. 2011;2:98. Epub 2011/01/01. doi: 10.3389/fimmu.2011.00098. PubMed PMID: 22566887; PubMed Central PMCID: PMCPMC3342348.

37. Federico A, Morgillo F, Tuccillo C, Ciardiello F, Loguercio C. Chronic inflammation and oxidative stress in human carcinogenesis. Int J Cancer. 2007;121(11):2381-6. Epub 2007/09/26. doi: 10.1002/ijc.23192. PubMed PMID: 17893868.

38. Lee YH, Han K, Ko SH, Ko KS, Lee KU. Data Analytic Process of a Nationwide Population-Based Study Using National Health Information Database Established by National Health Insurance Service. Diabetes Metab J. 2016;40(1):79-82. Epub 2016/02/26. doi: 10.4093/dmj.2016.40.1.79. PubMed PMID: 26912157; PubMed Central PMCID: PMCPMC4768054.

Grant support

There are no financial conflicts of interest to disclose.

Conflict of interest Disclosures

All authors have completed the conflict of interest disclosure form and declare that: (i) no support, financial or otherwise, has been received from any organization that may

have an interest in the submitted work; and (ii) there are no other relationships or activities that could appear to have influenced the submitted work

Author contributions

S.W.H. and H.J.L. contributed to manuscript drafting. S.W.H., H.J.L., and K.H. conceived the design of study and statistical analysis was performed. J.M.M., S.P., H.S., and E.A.K. contributed to the interpretation of results and revising the manuscript. J.C., J.P.I., and J.S.K. contributed the supervision and manuscript final editing.

Figure 1

Click here to download Figure Figure_1_300_TIFF.tiff

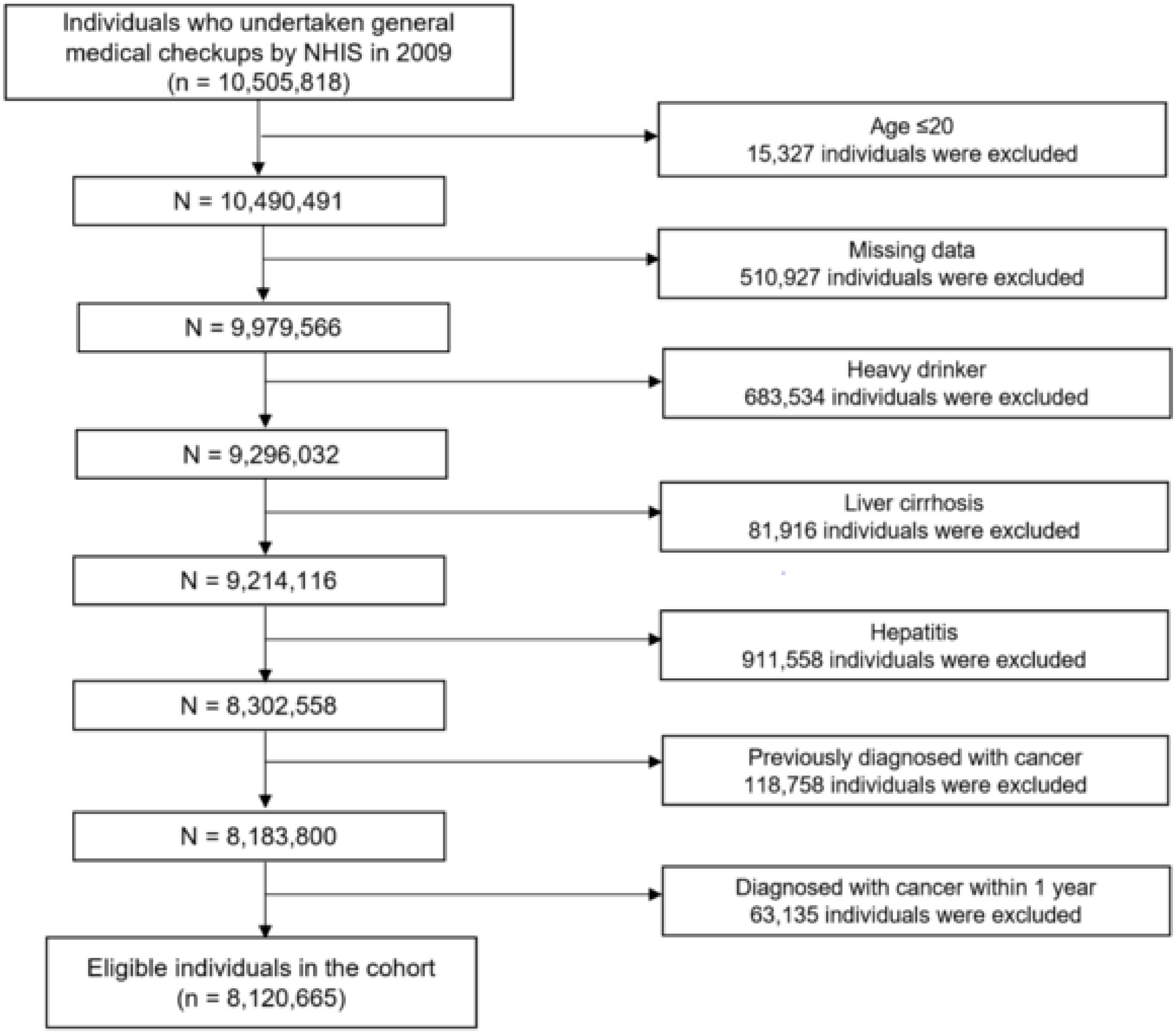

Figure 2

Click here to download Figure Figure_2_300_TIFF.tiff

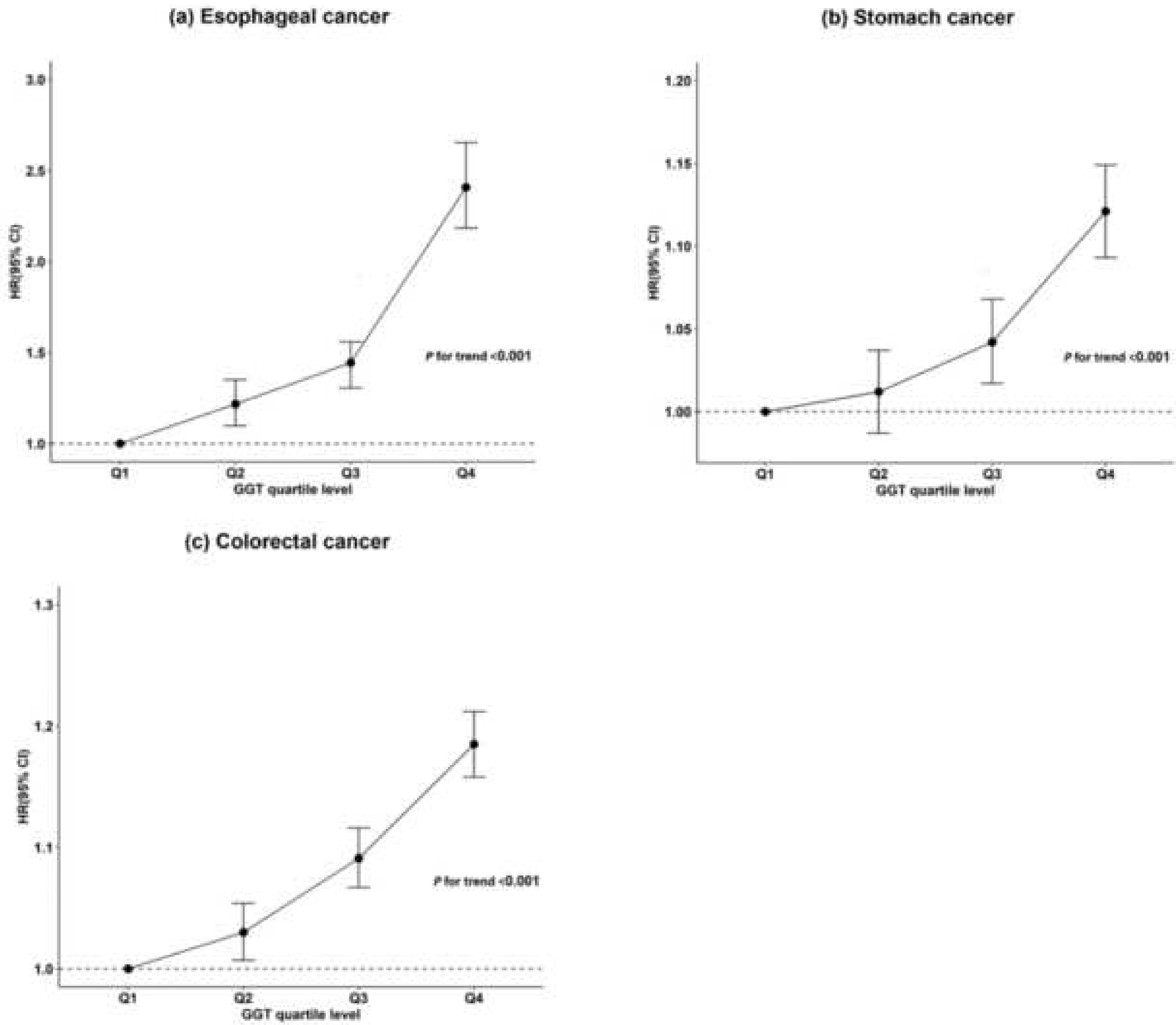

Figure 3

Click here to download Figure Fig.3_300_TIFF.tiff

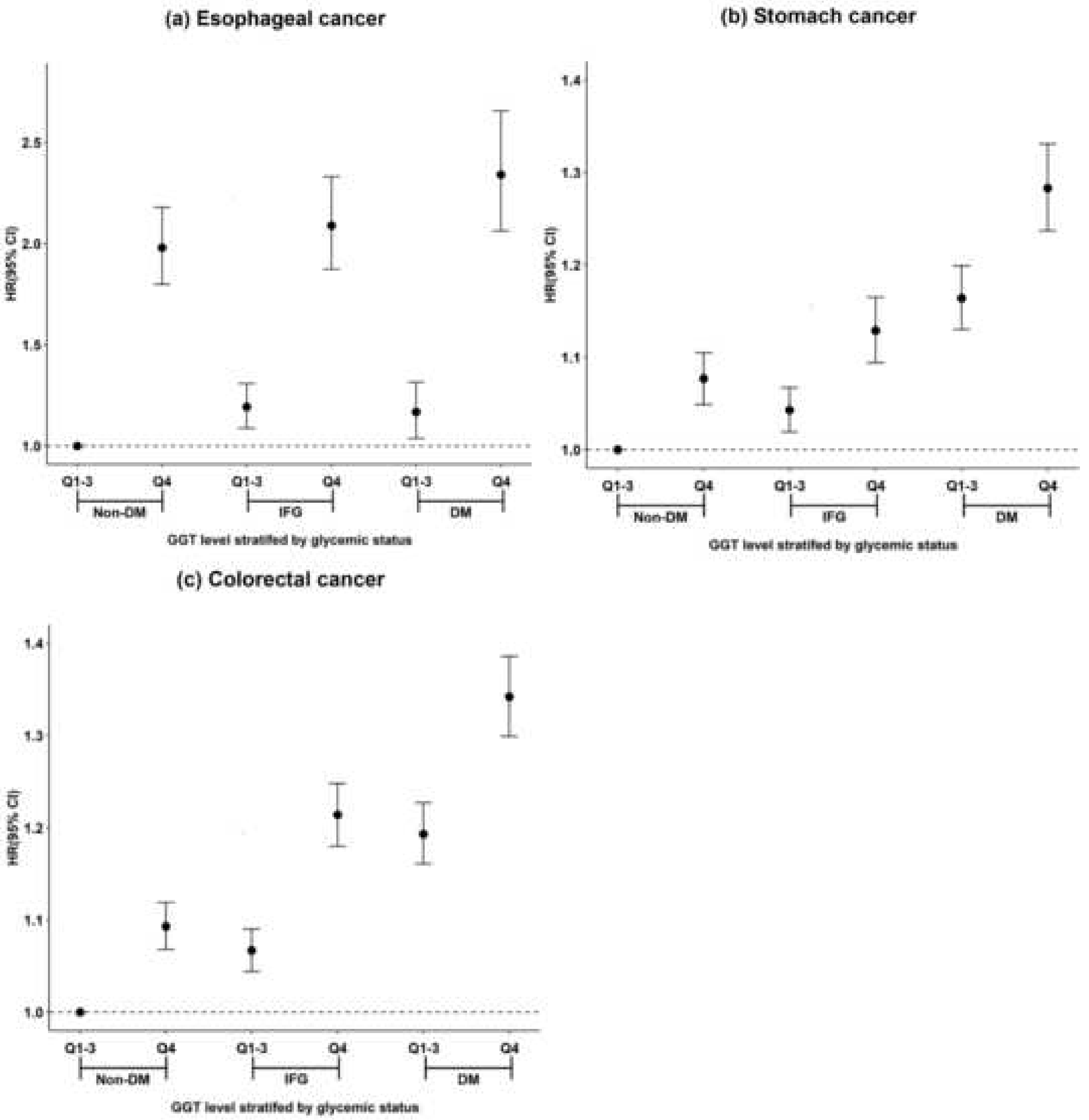

Supplement: Attachment — Submitted filename: PONE-D-20-12359_reviewer.pdf [file pone.0245052.s003.pdf]
