# Supplementary material for: Risk of gastrointestinal cancer in patients with an elevated level of gamma-glutamyltransferase: A nationwide population-based study
Source: PLoS One. 2021 Feb 5;16(2):e0245052. doi: 10.1371/journal.pone.0245052 (PMC7864398; doi:10.1371/journal.pone.0245052)
Supplement: Attachment — Submitted filename: comments_09272020.docx [file pone.0245052.s004.docx]

**Review for PLoS ONE**

**Sept. 27, 2020**

**Title:** Risk of Gastrointestinal Cancer in Patients with an Elevated Level of Gamma-glutamyltransferase: A Nationwide Population-Based Study

This paper presents a study assess whether an elevated serum gamma-glutamyltransferase (GGT) level is associated with the risk of gastrointestinal cancer through a large-scale study using nationwide data. In addition, we evaluated the effect of glycemic status on the association between an elevated GGT level and the risk of gastrointestinal cancer, and the contribution of the interaction between these two factors on the incidence of gastrointestinal cancer. However, there are questions that limit my enthusiasm of the paper, as outlined below.

1. Data collection and study population:
   a. What is the category for drinker? Is it heavy vs not-heavy?
   b. Why did authors consider the quartile level to categorize the GGT level? Instead of four categories, while not only consider two categories < Q4 and > Q4 (e.g., Table 4)? Using any threshold to categorize data can have considerable effect on the distribution of data. Please clarify this part.
2. Statistical method:
   a. Authors mentioned the t-test was done, but I couldn’t follow across the manuscript for which analysis the t-test was considered. Please clarify that part along with the more details about being two-sided or one-sided? Equal or unequal variance?
   b. Why two Cox models were fitted under each analysis? In addition to that did authors compare the two fitted Cox models using analysis of deviance?
   c. BMI variable wasn’t introduced in data collection and just used its abbreviation at method section.
3. Results:
   a. Table 1: Why not adding similar table as Suppl. To stratify GGT Q1-4 by sex (M/F)? I assume the reported P value is based on ANOVA test (continuous variables) and Chi-squared test (categorical). I could follow for which test t-test was done? (back to comment 2(a))
   b. Table 2, what is the reported P for trend?
   c. Instead of reporting P < 0.001, why not to report the exact P value using scientific notation?
