# Supplementary material for: Risk of gastrointestinal cancer in patients with an elevated level of gamma-glutamyltransferase: A nationwide population-based study
Source: PLoS One. 2021 Feb 5;16(2):e0245052. doi: 10.1371/journal.pone.0245052 (PMC7864398; doi:10.1371/journal.pone.0245052)
Supplement: Attachment — Submitted filename: Response to Reviewers_1104.docx [file pone.0245052.s005.docx]

**RE: PONE-D-20-12359**

Dear Editor-in-Chief

We are submitting our revised manuscript entitled “**Risk of Gastrointestinal Cancer in Patients with an Elevated Level of Gamma-glutamyltransferase: A Nationwide Population-Based Study**” for possible publication in the *PLOS ONE*.

We appreciate the valuable and insightful comments from the reviewers. We responded to each of the comments made and incorporated all modifications suggested by the reviewers into the revised manuscript. Please let us know if you have any further suggestion for changes.

Sincerely,

Hyun Jung Lee, MD, PhD.

E-mail: guswjd80@gmail.com

**Authors’ Response to the Reviewers’ Comments**

We thank the reviewers for their thoughtful and expert review of our manuscript and for their valuable and insightful comments. We responded to each of the comments made and incorporated all modifications suggested by the reviewers into the revised manuscript. Changes to the revised manuscript have been highlighted (underlined and in blue). Our responses to the Reviewers’ comments are as follows:

**Reviewer #2**

The manuscript is a good scientific sound. In addition, the manuscript can enplane more results and figures to show the behavior of data. Please, see the attached file, I put my comments on it.

1. Please, make more references.

**Reply:>**

Thank you for your comment. We added the references as you suggested in the Introduction and Discussion (Ref. No. [4], [16], and [41]).

**Reviewer #3**

This paper presents a study assess whether an elevated serum gamma-glutamyltransferase (GGT) level is associated with the risk of gastrointestinal cancer through a large-scale study using nationwide data. In addition, we evaluated the effect of glycemic status on the association between an elevated GGT level and the risk of gastrointestinal cancer, and the contribution of the interaction between these two factors on the incidence of gastrointestinal cancer. However, there are questions that limit my enthusiasm of the paper, as outlined below.

1. Data collection and study population:

a. What is the category for drinker? Is it heavy vs not-heavy?

**Reply:**

We really appreciate your comment. It is widely accepted that alcohol consumption is significantly associated with an elevated level of GGT. To minimize the confounding effect of excessive alcohol consumption on the serum GGT level, we defined those who drink more than 30g of alcohol per week as a heavy drinker and excluded them from the analysis. This was described in the subsection named “Data collection and study population” of the pre-revised manuscript (Page 5, line 77-79). Therefore, the category for a drinker was based on whether drink or not except for a heavy drinker.

b. Why did authors consider the quartile level to categorize the GGT level? Instead of four categories, while not only consider two categories < Q4 and > Q4 (e.g., Table 4)? Using any threshold to categorize data can have considerable effect on the distribution of data. Please clarify this part.

**Reply:**

Thank you for your comment. Previous several studies reported that the level of serum GGT in men generally is higher than that in women. Also, since this study is a nationwide population-based study, it cannot unify the measurement equipment, which could be attributed to variability in measured GGT levels. In addition, there is a lack of data about the GGT level, which is a threshold associated with an increased incidence of gastrointestinal cancer. Given these facts, we aimed to show the tendency of an increased risk of gastrointestinal cancer as the serum GGT level arises by dividing the population in accordance with the quartile level of GGT. Other studies regarding the clinical significance of a GGT level had also used the classification similar to our study. We have now added the following to the Materials and methods (Page 5, lines 81-82): “Since there might be a variability of GGT levels depending on measurement equipment and no threshold was reported to be associated with an increased risk of gastrointestinal cancer,…..”. Also we added the references in the Introduction.

2. Statistical method

a. Authors mentioned the t-test was done, but I couldn’t follow across the manuscript for which analysis the t-test was considered. Please clarify that part along with the more details about being two-sided or one-sided? Equal or unequal variance?

**Reply:**

Thank you for your detailed comment. We reviewed the method of analysis with the statisticians, co-author of this study, and found that as you pointed out, we had never used the t-test in our analysis. We deleted the mention of the t-test in the revised manuscript. Meanwhile, we assumed equal variance after a review by the statisticians and used one-sided ANOVA, which is now modified in the Materials and methods (Page 6, lines 105-106): “To compare differences among groups, one-sided ANOVA were used for…”.

b. Why two Cox models were fitted under each analysis? In addition to that did authors compare the two fitted Cox models using analysis of deviance?

**Reply:**

Thank you for your high-quality comment. The aim of this study was to reveal the association between an elevated level of GGT and the risk of gastrointestinal cancer. To clarity this association, we implemented the hierarchical regression analysis, including variables which were known to be risk factors for gastrointestinal cancer. Therefore, we presented the first model with age and sex and, another model with additional variables including BMI, smoking, drinking, exercise, and income, which is now added to the Materials and methods (Page 7, lines 112-113): “… which were known to be risk factors for gastrointestinal cancer.”. Whereas, we focused on the association itself between an elevated GGT level and the risk of gastrointestinal cancer, not on which model was suitable to explain the association. Thus, we did not compare the quality of models through an analysis of deviation.

c. BMI variable wasn’t introduced in data collection and just used its abbreviation at method section.

**Reply:**

I am grateful for your comment. Body mass index (BMI) is a calculation using a person’s height and weight. The formula is BMI = kg/m^2^ where kg is a person's weight in kilograms and m^2^ is their height in meters squared. We added the description regarding BMI in the Materials and method (Page 4, lines 64-65): “Body mass index (BMI) was by dividing the weight (kg) by height squared (m^2^).”.

3. Results:

a. Table 1: Why not adding similar table as Suppl. To stratify GGT Q1-4 by sex (M/F)? I assume the reported P value is based on ANOVA test (continuous variables) and Chi-squared test (categorical). I could follow for which test t-test was done? (back to comment 2(a))

**Reply:**

We appreciate your splendid suggestion. As you suggested, we added the supplementary table presenting baseline characteristics according to the GGT level after dividing by sex. (Page 7, lines 125-127): “When the baseline characteristics were analyzed upon stratification by sex, the results showed similar trend in both men and women according to the GGT quartile level (S1 and S2 table).” As we mentioned in comment 2a, we didn’t use the t-test in the study and modified in the Materials and methods (Page 6, lines 104-105). Thank you again for your point.

b. Table 2. what is the reported P for trend?

**Reply:**

We are thankful for your comment. The p-value reported in Table 2 indicated a statistical significance for the linear increase. In other words, the greater degree of increase in GGT, the greater risk of gastrointestinal cancer. In addition, we conducted a trend analysis on the association between glycemic status and risk of gastrointestinal cancer in Table 3. As a result, the risk of gastrointestinal cancer significantly increased as the glycemic status progressed, regardless of the type of cancer. We added the statistical results in Table 3.

c. Instead of reporting P < 0.001, why not to report the exact P value using scientific notation?

**Reply:**

Thank you for your comment. As far as we know, the American Psychological Association style manual recommended that when reporting *p* values, the authors should report exact *p* values to two or three decimal places. However, it recommended to report *p* values less than .001 as *p* < .001. As recommended in the manual, we stated the *p* value as *p* < .001 in case of *p* values less than .001. (Fidler, Fiona. "The American Psychological Association publication manual sixth edition: implications for statistics education." Proceedings of the Eighth International Conference on Teaching Statistics (ICOTS8. 2010.) p160)
